# Pregnancy Outcomes of Mothers with Detectable CMV-Specific IgM Antibodies: A Three-Year Review in a Large Irish Tertiary Referral Maternity Hospital

**DOI:** 10.1155/2015/218080

**Published:** 2015-11-30

**Authors:** Richard J. Drew, Patrick Stapleton, Hala Abu, Eibhlín Healy, Wendy Ferguson, Cillian De Gascun, Joanne O'Gorman, Maeve Eogan

**Affiliations:** ^1^Rotunda Hospital, Parnell Square, Dublin 1, Ireland; ^2^Royal College of Surgeons in Ireland, Dublin 2, Ireland; ^3^National Virus Reference Laboratory, University College Dublin, Dublin 4, Ireland; ^4^School of Medicine, University College Dublin, Dublin 4, Ireland

## Abstract

A retrospective audit was performed for all obstetric patients who had positive CMV IgM results between January 2012 and December 2014 in the Rotunda Hospital, Ireland. In total, 622 CMV IgM positive tests were performed on samples from 572 patients. Thirty-seven patients had a positive CMV IgM result (5.9%) on the Architect system as part of the initial screening. Three patients were excluded as they were not obstetric patients. Of the 34 pregnant women with CMV IgM positive results on initial screening, 16 (47%) had CMV IgM positivity confirmed on the second platform (VIDAS) and 18 (53%) did not. In the 16 patients with confirmed positive CMV IgM results, four (25%) had acute infection, two (12.5%) had infection of uncertain timing, and ten (62.5%) had infection more than three months prior to sampling as determined by the CMV IgG avidity index. Two of the four neonates of women with low avidity IgG had CMV DNA detected in urine. Both these cases had severe neurological damage and the indication for testing their mothers was because the biparietal diameter (BPD) was less than the 5th centile at the routine 20-week gestation anomaly scan.

## 1. Introduction

Congenital CMV (cCMV) can have devastating consequences for affected infants and their families, as infection can lead to deafness, severe neurological impairment, and also learning difficulties. A major systematic review of sensorineural hearing loss showed that 10–20% of cases were due to congenital CMV, and thus, additionally, it has a significant health economic impact [1]. Recently Kimberlin and colleagues reported a benefit associated with early antiviral treatment of cCMV, in particular on hearing loss [2]. This treatment increases the need to have a robust screening method in place so that children infected with CMV at birth can be identified. Neonatal screening for CMV infection using the polymerase chain reaction (PCR) on urine or saliva has been shown to be cost effective if a targeted approach is employed [3–6]. Like most hospitals, we have traditionally operated a system of testing for CMV seroconversion in certain patient populations (e.g., second trimester miscarriage and intrauterine death or babies born to HIV-infected women or women on immunosuppression during pregnancy), but we do not have a general antenatal or neonatal screening policy.

A recent study in another maternity hospital in Dublin, Ireland, showed that the congenital CMV incidence was 0.19% by neonatal salivary testing [7]. Thus, it is possible to calculate what the expected number of infected infants would be in our institution and to examine how many of these cases were detected by our current testing programme. It is our hypothesis that very few cases of congenital CMV are detected by antenatal serology testing and that many infants are not identified in the neonatal period, thus missing out on the opportunity to receive antiviral treatment.

## 2. Materials and Methods

This was a retrospective audit of all obstetric patients who had positive CMV IgM results between January 2012 and December 2014 in the Rotunda Hospital, Dublin, Ireland. The aim was to determine the indications for the initial CMV IgM testing and also the neonatal outcome. A secondary aim was to determine what percentage of expected cases with congenital CMV was diagnosed through maternal serology. Based on the study of Waters and colleagues, our hospital would expect to have 51 (0.19% ×  26,862 live births >500 g) children with cCMV during the study period.

The Rotunda Hospital is specialist tertiary referral maternity hospital which had 26,862 live births >500 g recorded during the study period. The hospital serves a diverse population in terms of ethnicity, and in 2012 and 2013, 64% and 73%, respectively, of patients delivering were Irish. The sole inclusion criterion was a positive CMV IgM in an obstetric patient who was tested either during pregnancy or immediately after delivery (i.e., in the setting of miscarriage or intrauterine death). There were no exclusion criteria. Patients were followed up until delivery so that fetal outcome could be recorded. For mothers with confirmed detectable CMV-specific IgM, the results of urinary CMV DNA testing in the neonate were recorded. This was taken as confirmation of cCMV in the neonate, if performed in the first 21 days of life.

The positive CMV IgM results were identified following an electronic search of the Laboratory Information System at the Rotunda Hospital where the patients attended. The serology testing was performed at the National Virus Reference Laboratory in Ireland (NVRL). CMV IgM testing was initially performed on the Architect system (Abbott Diagnostics) and then confirmed on the VIDAS (BioMerieux, France). This was the standard algorithm that was in place at the time of the testing for all clinical specimens. Urinary CMV DNA testing was carried out using an Artus kit on the ABI 7500 FAST Real-Time PCR system. All tests were carried out in accordance with the manufacturers' instructions.

According to hospital guidelines, CMV IgM testing is performed in all cases where there are abnormal antenatal ultrasound findings, such as intrauterine growth restriction, echogenic bowel, microcephaly, and cardiac anomalies. It also typically requested in unexplained cases of sudden intrauterine death; in women with second trimester miscarriages; and in certain patients with transaminitis. The diagnostic criteria for CMV infection in the mother were taken to be confirmed CMV IgM positive result on two distinct assays (Architect and VIDAS) as above. Samples yielding positive results on the Architect that were not confirmed on the Vidas were excluded from the study. On confirmation of the CMV IgM result, CMV IgG avidity testing was performed (VIDAS, BioMerieux). A diagnosis of recent (defined as within three months of date of sample) CMV infection was made if the avidity index was ≤0.4. Patients with an avidity index of ≥0.65 were considered to have had infection more than three months prior to sampling, while patients with an equivocal avidity index (between 0.4 and 0.6) were deemed to have had CMV infection at some time, but the timing could not be determined.

In neonates, cCMV infection was taken to be confirmed if they had a CMV DNA detected by PCR in their urine in the first 21 days of life. For the purposes of this study, only urinary CMV DNA results were recorded for neonates born to mothers with positive CMV IgM results.

The data was recorded electronically in Microsoft Excel, and only descriptive statistics were performed. Chi-square statistics were performed to compare parity status and were performed using MedCalc software, version 15.6.1.

## 3. Results

During the study period from January 2012 to December 2014 there were 26,862 live births >500 g recorded. The breakdown of the CMV IgM results is shown in Table 1.   In total, 622 CMV IgM positive tests were performed on samples from 572 patients. There were 37 patients with a positive CMV IgM result (5.9%) on the Architect system as part of the initial screening. Three patients were excluded as they were not obstetric patients: of the 34 remaining, there were 32 singleton pregnancies and 2 twin pregnancies.

The median age of the 34 patients with detectable CMV IgM was 30 years, with an interquartile range of 25–34 years (range 19–43 years). Of the 34 patients, 22 were White Irish (65%), six (17%) were White non-Irish, two (6%) were non-White Asian, and four (12%) were unknown. The median gestation at testing was 28 weeks, with an interquartile range of 17 to 33 weeks (range 6–41 weeks). The gestation at delivery was available for 30 of 34 mothers, with a median gestation of 37 weeks and an interquartile range of 31–38 weeks (range 15–41 weeks). Seven (21.8%) of 32 women were nulliparous, and previous obstetric history was not available in two women. In comparison to hospital statistics for 2013, in which 3689 of 8648 (42.7%) women were nulliparous, there was a statistically significant higher rate of multiparity in CMV IgM positive women (*χ* ^2^ = 4.81, *P* = 0.02). The most common reason to consider CMV infection was intrauterine growth restriction (IUGR) (*n* = 6) seen on antenatal scan, followed by raised liver enzymes (*n* = 5) and intrauterine death (*n* = 4) (Table 2).

The clinical outcome of the babies of the 34 women is shown in Table 1.   Of these 34 patients, 16 (47%) had CMV IgM positivity confirmed on the second platform (VIDAS) and 18 (53%) did not. Of the 16 patients with confirmed positive CMV IgM results, four (25%) had acute infection, two (12.5%) had infection of uncertain timing, and ten (62.5%) had infection more than three months prior to sampling as determined by the CMV IgG avidity index.

Two of the four neonates of women with low avidity IgG had CMV DNA detected in urine. Both these cases had severe neurological damage and the indication for testing their mothers was because the biparietal diameter (BPD) was less than the 5th centile at the routine 20-week gestation anomaly scan. In the two cases of mothers with low CMV IgG avidity, but no CMV DNA detected in the neonatal urine, both fetuses had BPDs above the 30th centile at the 20-week anomaly scan. The indications for testing in the mothers of these two unaffected infants were maternal request, and asymmetrical IUGR was noted on routine antenatal ultrasound at 31 weeks gestation. Three of the four neonates born to mothers with high CMV IgG avidity had urinary CMV PCR tests performed, and all were negative in these healthy live-born infants.

## 4. Conclusion

This study has shown that over a three-year period in which there were 26,862 live births >500 g, assuming an Irish cCMV incidence of 0.19% [7], then only two of the expected 51 congenitally infected infants were identified over the study period. It is notable that the two mothers of the congenitally infected infants both tested IgM positive in the first 15 weeks of pregnancy. This emphasises the fact that as first trimester acquisition of CMV is usually most severe, early infection in pregnancy is most likely to be detected on the basis of a clinically directed targeted screening approach as is currently the case in our hospital.

Two key limitations of this study are that it is retrospective and that the indications for testing are biased by testing guidelines. The recommendation is to screen for congenital CMV in second trimester miscarriage, for example. It would be interesting to study these patients in future to try to determine the role, if any, that CMV had in the miscarriage or the intrauterine death. As a result there may be an overrepresentation of intrauterine deaths in the patients studied. In addition, two mothers in the study who had confirmed CMV IgM positive results were lost to follow-up and it is not clear what the neonatal outcome was in those patients. During the study period CMV IgM immunoblot testing was not performed to definitively categorise cases and this could be done as part of future studies to help in determining whether cases represented new infection or reactivation. As this was a retrospective study, the testing algorithm that was in place at the time was used, although there may be other testing algorithms now available for use. Also repeat CMV IgG testing was not performed in all patients to demonstrate seroconversion.

The results shown in this study demonstrate that an alternative to the current approach is required if all, or at least a greater proportion of, cases of cCMV are to be identified in the neonatal period, allowing for early antiviral treatment if required. The low detection rate for cCMV cases described herein is disappointing. Universal urinary or salivary testing for CMV DNA in neonates in the postnatal wards would increase the rate of identification of cCMV cases.

Caution would have to be used when trying to generalise these results to other populations, as it would be necessary to take account of background CMV seropositivity and also ethnicity. CMV seroprevalence rates in pregnant women in Ireland are known to be low by international standards [7]. As the treatment of cCMV improves it is more important that neonates are identified early and that any hearing loss can be reduced or minimsed. The present approach to cCMV detection is suboptimal, and there is a need to examine other potential screening methods such as neonatal urine/salivary CMV DNA testing.

## Figures and Tables

**Table 1:** Overview of the CMV IgM positive results in patients at the Rotunda Hospital between 2012 and 2014.

622 CMV IgM tests sent from 572 patients			

↓		↓	

37 patients with positive CMV IgM (Architect)		535 patients with negative CMV IgM	

↓			

34 obstetric patients included in analysis 3 nonobstetric patients removed			

↓			

CMV IgM confirmed on second platform (Vidas)?		→	No (*n* = 18)

↓			↓

Yes (*n* = 16)			↓

↓	↓	↓	↓

**Avidity ≤ 0.4** (Acute infection <3 months ago)	**Avidity >0.4 and <0.65** (Date of infection uncertain)	**Avidity ≥0.65** (Previous infection acquired > 3 months ago)	**Cross reactivity** (CMV IgM positivity not confirmed in 2 assays)

↓	↓	↓	↓

*N* = 4	*N* = 2	*N* = 10	*N* = 18

↓	↓	↓	↓

*N* = 2, severe laboratory confirmed congenital CMV disease with neurological abnormalities (maternal CMV IgM tests sent at 11 and 15 weeks' gestation, resp.) *N* = 1, IUGR but CMV not detected in urine at birth (maternal CMV IgM test sent at 30 weeks) *N* = 1, normal but CMV not detected in urine at birth (maternal CMV IgM test sent at 31 weeks' gestation)	*N* = 1, intrauterine death *N* = 1, lost to follow-up	*N* = 4, healthy live-born (3 neonates did not have CMV detected in urine, 1 was not tested) *N* = 3, stillborn *N* = 1, multiple congenital anomalies detected in utero and lost to follow-up *N* = 1, early neonatal death due to prematurity at 26 wk gestation (twins) *N* = 1, miscarriage	*N* = 11, healthy live-born pregnancies (1 set of twins) *N* = 3, Intrauterine death *N* = 2, miscarriage *N* = 2, went abroad for delivery

**Table 2:** Indications given for requesting the maternal CMV IgM test.

Reason for test	Number	Percentage
Intrauterine growth retardation	6	18%
Raised liver enzymes	5	15%
Intrauterine death	4	12%
Miscarriage	3	9%
Not recorded	3	9%
Fetal pleural effusion	2	6%
Stillborn	2	6%
Abnormal scan (not specified)	1	3%
Congenital anomaly (cardiac)	1	3%
Echogenic bowel	1	3%
Lethargy	1	3%
Microcephaly	1	3%
Oligohydramnios	1	3%
Previous child had CMV	1	3%
Polyhydramnios	1	3%
Maternal request	1	3%
